# Supplementary material for: MetaQTL: a package of new computational methods for the meta-analysis of QTL mapping experiments
Source: BMC Bioinformatics. 2007 Feb 8;8:49. doi: 10.1186/1471-2105-8-49 (PMC1808479; doi:10.1186/1471-2105-8-49)
Supplement: Additional File 7 — MetaQTL Package : jar file and tutorial. This Zip archive contains both the MetaQTL JAR file and the files of the tutorial. [file 1471-2105-8-49-S7.zip › org.inra.metaqtl/doc/overview-frame.html]

Overview


All Classes

Packages
  
org.metaqtl
  
org.metaqtl.adapter
  
org.metaqtl.algo
  
org.metaqtl.bio
  
org.metaqtl.bio.entity
  
org.metaqtl.bio.entity.adapter
  
org.metaqtl.bio.entity.bean
  
org.metaqtl.bio.entity.factory
  
org.metaqtl.bio.util
  
org.metaqtl.factory
  
org.metaqtl.graph
  
org.metaqtl.main
  
org.metaqtl.numrec
  
org.metaqtl.util
